# Assessing health research grant applications: A retrospective comparative review of a one-stage versus a two-stage application assessment process

**DOI:** 10.1371/journal.pone.0230118

**Published:** 2020-03-12

**Authors:** Ben Morgan, Ly-Mee Yu, Tom Solomon, Sue Ziebland

**Affiliations:** 1 National Institute for Health Research Central Commissioning Facility, Twickenham, England, United Kingdom; 2 Nuffield Department of Primary Care Health Sciences, University of Oxford, Oxford, England, United Kingdom; 3 Faculty of Health and Life Sciences, University of Liverpool, Liverpool, England, United Kingdom; University of Toronto, CANADA

## Abstract

**Background:**

Research funders use a wide variety of application assessment processes yet there is little evidence on their relative advantages and disadvantages. A broad distinction can be made between processes with a single stage assessment of full proposals and those that first invite an outline, with full proposals invited at a second stage only for those which are shortlisted. This paper examines the effects of changing from a one-stage to a two-stage process within the UK’s National Institute for Health Research’s (NIHR) Research for Patient Benefit (RfPB) Programme which made this change in 2015.

**Methods:**

A retrospective comparative design was used to compare eight one-stage funding competitions (912 applications) with eight two-stage funding competitions (1090 applications). Comparisons were made between the number of applications submitted, number of peer and lay reviews required, the duration of the funding round, average external peer review scores, and the total costs involved.

**Results:**

There was a mean number of 114 applications per funding round for the one-stage process and 136 for the two-stage process. The one-stage process took a mean of 274 days and the two-stage process 348 days to complete, although those who were not funded (i.e. the majority) were informed at a mean of 195 days (mean 79 days earlier) under the two-stage process. The mean peer review score for full applications using the one-stage process was 6.46 and for the two-stage process 6.82 (5.6% difference using a 1–10 scale (with 10 being the highest), but there was no significant difference between the lay reviewer scores. The one-stage process required a mean of 423 peer reviews and 102 lay reviewers and the two-stage process required a mean of 208 peer reviews and 50 lay reviews (mean difference of 215 peer reviews and 52 lay reviews) per funding round. Overall cost per funding round changed from £148,908 for the one-stage process to £105,342 for the two-stage process saving approximately £43,566 per round.

**Conclusion:**

We conclude that a two-stage application process increases the number of applications submitted to a funding round, is less burdensome and more efficient for all those involved with the process, is cost effective and has a small increase in peer reviewer scores. For the addition of fewer than 11 weeks to the process substantial efficiencies are gained which benefit funders, applicants and science. Funding agencies should consider adopting a two-stage application assessment process.

## Introduction

There are many different funders of research who operate funding schemes ranging from small individual projects to large infrastructure awards. In the UK alone approximately £3bn was spent on health relevant research in 2014[1] and US$ 6.5 trillion was spent worldwide in 2012[2]. It is common practice before grants are awarded for some sort of assessment to take place, often involving peer review by external experts and/or an expert panel review. The specific commissioning process used to select the applications to be funded will typically be decided by the funder reflecting the size, scale and importance of the funding being allocated. However, there is little evidence or data to help funders decide the most appropriate commissioning process to balance the amount of money spent *on* research with the cost of the assessment process itself, including the actual or opportunity costs for all involved. Researchers in Australia have shown that a substantial amount of time and effort goes into preparing proposals, 34 days on average, and that shortened applications processes could reduce lost research output[3]. A Canadian study also estimated that the cost of preparing a research grant application amounted to $40,000 (Canadian) and it could be more appropriate to provide direct grants to qualified researchers to improve research output[4]. The National Health and Medical Research Council (NHMRC) of Australia has conducted a number of studies looking at different ways to assess research proposals including simplified processes with the potential to save time and money without detracting from the rigour of the process. The NHMRC also showed that a more streamlined application process with accelerated peer review saved time for applicants and peer reviewers[5]. The National Science Foundation (NSF) adopted a two-stage application process which increased the total number of applications. Although the new NSF process resulted in a reduction in the overall success rate it increased the success rate for the second stage full applications[6]. While there are generally few studies on the overall research application assessment processes a related area, looked at in some detail, is the value of external peer review[7–10] where previous research has examined the optimal numbers and expertise required. Guthrie et al point to the lack of evidence on the efficiency of peer review, where there may be biases in the systems used by funders and substantial burden to those involved, notably applicants[11].

Therefore, the operational benefits of particular commissioning processes are not routinely reviewed or discussed and very few funding programmes have reported evidence about the effects of changed processes. New funding programmes being developed, and established programmes reviewing their processes, may wish to consider whether there are any benefits to a two-stage assessment process. Public sector research funders, in the interests of transparency, should also review their process and make more data accessible as described in the NIHR Adding Value in Research framework[12].

### About the National Institute for Health Research (NIHR)

The NIHR, established in 2006 and funded by the UK’s Department of Health and Social Care, is the UK’s largest funder of health and care research spending approximately £1.1b annually[13]. The NIHR’s mission is to improve the health and wealth of the nation through research, which it achieves by funding a variety of research in various formats. These include research Infrastructure such as the Biomedical Research Centres and the support to deliver research through the Clinical Research Network. There is substantial investment in training and research fellowships at all levels from pre-doctoral to senior investigators. The NIHR Research programmes are a sizeable investment in research receiving approximately a quarter of the overall budget. The programmes have different remits and eligibility[14]. Most of the NIHR programmes now operate a two-stage application assessment process. The NIHR’s Research for Patient Benefit (RfPB) programme changed from a one-stage to two-stage assessment process in 2015.

One of the strengths of the NIHR is its use of patients and the public as lay reviewers throughout the whole application assessment process to ensure patients’ views are considered when deciding which research to fund. Lay reviewers are members of the public, patients and/or carers who provide a different perspective in addition to the researchers and clinicians. Lay reviewers will often assess whether a research application is relevant to patients, adequately includes patients as part of the research, has sensible recruitment processes and is not too burdensome for participants[15].

### About the NIHR RfPB programme

The NIHR RfPB programme is a UK health and care research programme that funds individual research projects up to the value of £350k, assessed by 8 regional panels in England. The programme received over 5000 applications from researchers between its first funding round in 2006 and 2017. The programme holds three researcher-led funding rounds per year which typically receive between 100 to 150 applications each funding round. As a research programme receiving a high volume of applications it is important the application assessment processes are efficient, proportionate, acceptable to those involved in the process and support strong research. The RfPB programme’s recent history offers a good opportunity to explore the benefits of a two-stage application process due to the volume of applications and the high number of panel meetings and external reviewers involved.

Although receiving a relatively high number of applications initially (2006), over time the number of applications to the programme steadily decreased to the point where (in 2014) the programme received 82 applications and operated more panel assessing capacity than needed, occasionally resulting in the cancellation of panel meetings due to lack of applications to assess i.e. only eight or nine of the ten regional panels were needed. From the applicants’ perspective it was essentially an ‘all or nothing’ system; the one-stage process could be seen as requiring disproportionate effort for a funding cap of £350k. The one-stage process did not offer the applicants opportunity to address any feedback from either the panel or external peer/lay reviewers and applicants had to wait a relatively long time to find out if their applications were funded.

This paper assesses the effect of changes to the research application assessment process of the National Institute for Health Research’s (NIHR) Research for Patient Benefit (RfPB) programme which in 2015 moved from a one stage to two-stage application assessment process. We wanted to find out whether the two-stage process was more appropriate, efficient, cost effective and supportive of high quality science for the RfPB programme, which receives a high number of applications.

## Methods

Data held on the RfPB databases were extracted in December 2018 between funding round 20 and funding round 35, which ran from 2013 to 2018. These data were all stored on RfPB’s Research Management System (RMS). Data were extracted into a Microsoft Excel sheet and checked against the RMS and other programme statistics for accuracy. These 16 funding rounds comprised the eight most recent completed funding rounds using the two-stage application process, and the last eight funding rounds of the one-stage commissioning process.

The following areas were compared between the single and two-stage commissioning processes:

1. Assessment of the overall timeframe of the commissioning processes from application submission to notification of the outcome
2. Any changes in the quality of the science within the applications, using the external peer and lay review scores as an indicator of quality
3. Observation of any changes in the number of applications submitted to the programme and effect on success rates
4. Assessment of the number of peer reviewers required to facilitate each applications process
5. Assessment of any change to the costs of the new process

Analysis was primarily descriptive. Data were summarised in frequency for count data, and mean and standard deviation for continuous data. Comparison of duration of funding round between the two processes was performed using unpaired t-test. A mixed effect model was used to compare the mean review scores between the two processes, adjusting the clustering of reviewers within the submitted applications which was included as a random effect. Process (i.e. one-stage or two-stage), reviewers, and type of review (lay or expert review) were fitted as fixed effect. A process by reviewer type interaction was also fitted to assess whether the difference in the reviewers scores between the process were dependent on the type of reviewers. Analysis was performed using Stata SE version 15.1[16]

### Description of the one-stage and two-stage processes

In 2015 the RfPB programme adopted a two-stage application process with the intention of making the assessment process more efficient and proportionate for all involved: research applicants, external peer/lay reviewers, panel members, and the funder. In addition to this change RfPB also reduced its number of regional panels from ten to eight based on the view that a more efficient process would require less panel assessing capacity. Without changing to a two-stage process it was considered too high risk to reduce the number of panels because the one-stage process was less flexible when dealing with a high volume of applications. When developing the two-stage process RfPB anticipated that it would be able to invite approximately 50% of outline applications to full applications and then fund approximately 50% of the full applications at stage 2.

In implementing this change to a two-stage process the intentions were to:

1. Assess applications within a similar timeframe to the previous one-stage process
2. Maintain or improve the quality of the science
3. Maintain or increase the number of applications received
4. Reduce the number of external peer and lay reviews sought
5. Increase cost efficiency

The one-stage process involved each panel of around 15–20 senior methodologists and clinical researchers, including two to three lay members, assessing an average of 10–15 applications during a (sometimes long) one day meeting. There were three funding rounds a year and three rounds of panel meetings where the 10 regional panels assessed applications. Therefore, each year there were 30 panels meetings held by the programme.

**Fig 1:**
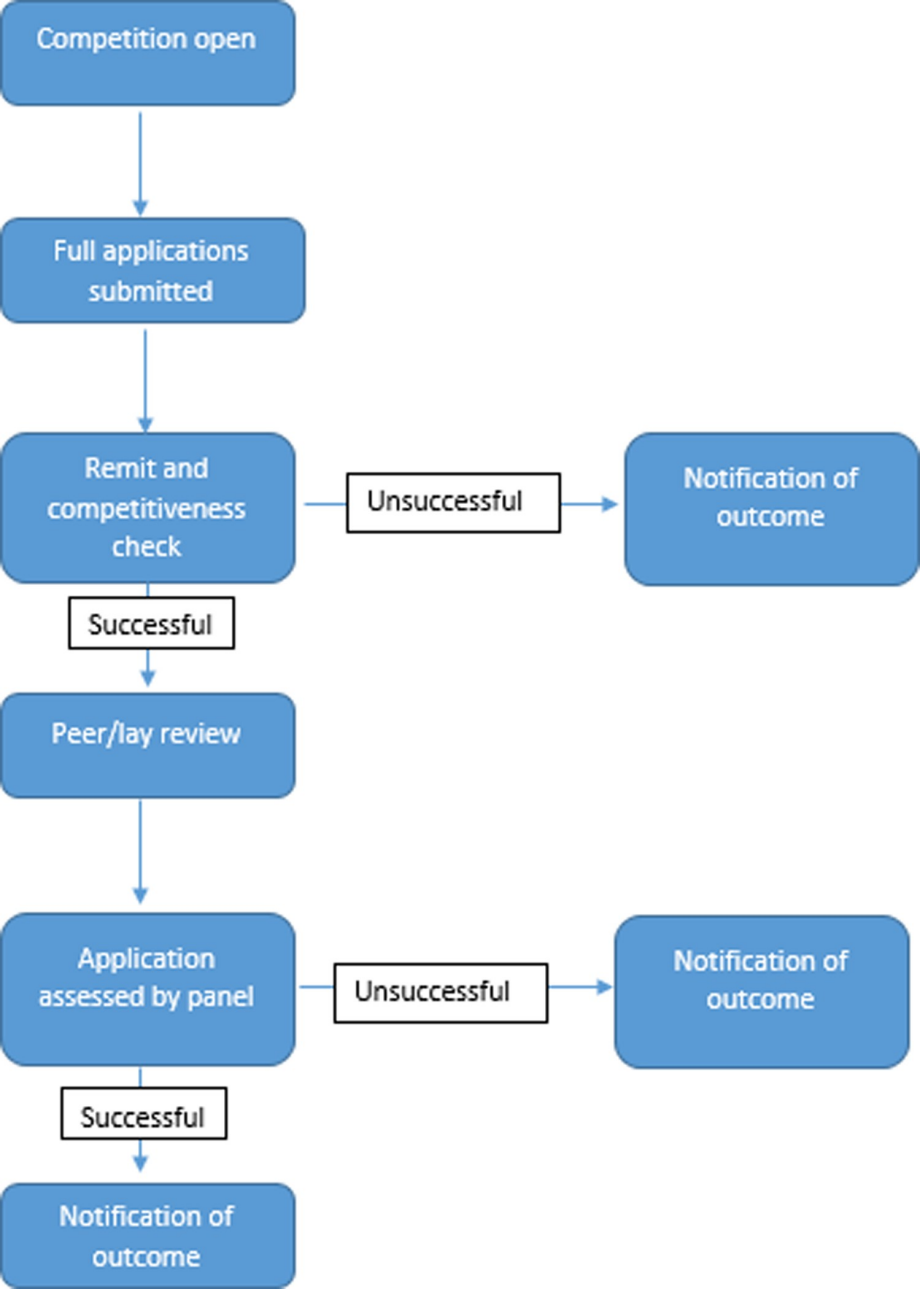
Flowchart of one-stage assessment process.

The one-stage process, as shown in Fig 1, required all applicants to complete a full application form. All full application forms submitted under the one-stage process were initially reviewed by the RfPB staff and panel chairs for remit and competitiveness checks, essentially acting as a triage to help manage workload. The remit and competitive checks were a relatively swift triage for applications which were out of the RfPB remit and/or were incomplete or clearly uncompetitive. All full applications which passed the remit and competiveness checks were then sent for external peer and lay review. The peer reviewers were typically methods and topic experts who did not currently sit on an RfPB panel. The RfPB panel then assessed the full applications, factoring in comments from the external peer and lay reviewers, to decide whether or not to recommend the applications for funding. When making their decisions the RfPB panels use the following broad criteria:

• scientific quality of the proposal
• the likely impact on health and social care and the potential for patient benefit in the short to medium-term
• the value for money provided by the proposal

The panels then scored each application using a 1 to 10 range (with 10 being the highest) and used the mean score to rank the applications and decide which applications to recommend for funding.

The one-stage application form asked questions about all aspects of the proposal including the background, aims and objectives, research plan, dissemination, finances, intellectual property, management and governance as well as other sections. The mandatory sections of the one-stage form had a collective word limit of (up to) 10,000 words. Applications ranged in size due to attached appendices, however applications usually ranged between 50 to 100 pages. Only those that were clearly out of scope on a competitiveness and remit check avoided full review.

The two-stage process similarly involves panels of around 15–20 senior methodologists and clinical researchers, including two to three lay members, assessing a number of applications submitted to the programme. Although there were still three funding calls held each year and three rounds of panel meetings, the reduction from ten to eight panels meant that only 24 meetings were held. Despite having two stages of panel assessment the programme configured the panel meetings so that, a single, one day panel meeting can assess both the full applications from one funding round and the outline applications for the subsequent round (e.g. the full applications of competition 30 and outline applications of competition 31 would have been assessed during a single, one day panel meeting). This means that for three funding rounds a year only 24 panel meetings are required for the two-stage process with eight panels. The two-stage process also allowed applications invited to submit a full application to defer to the following funding round to provide more flexibility to the research teams.

**Fig 2:**
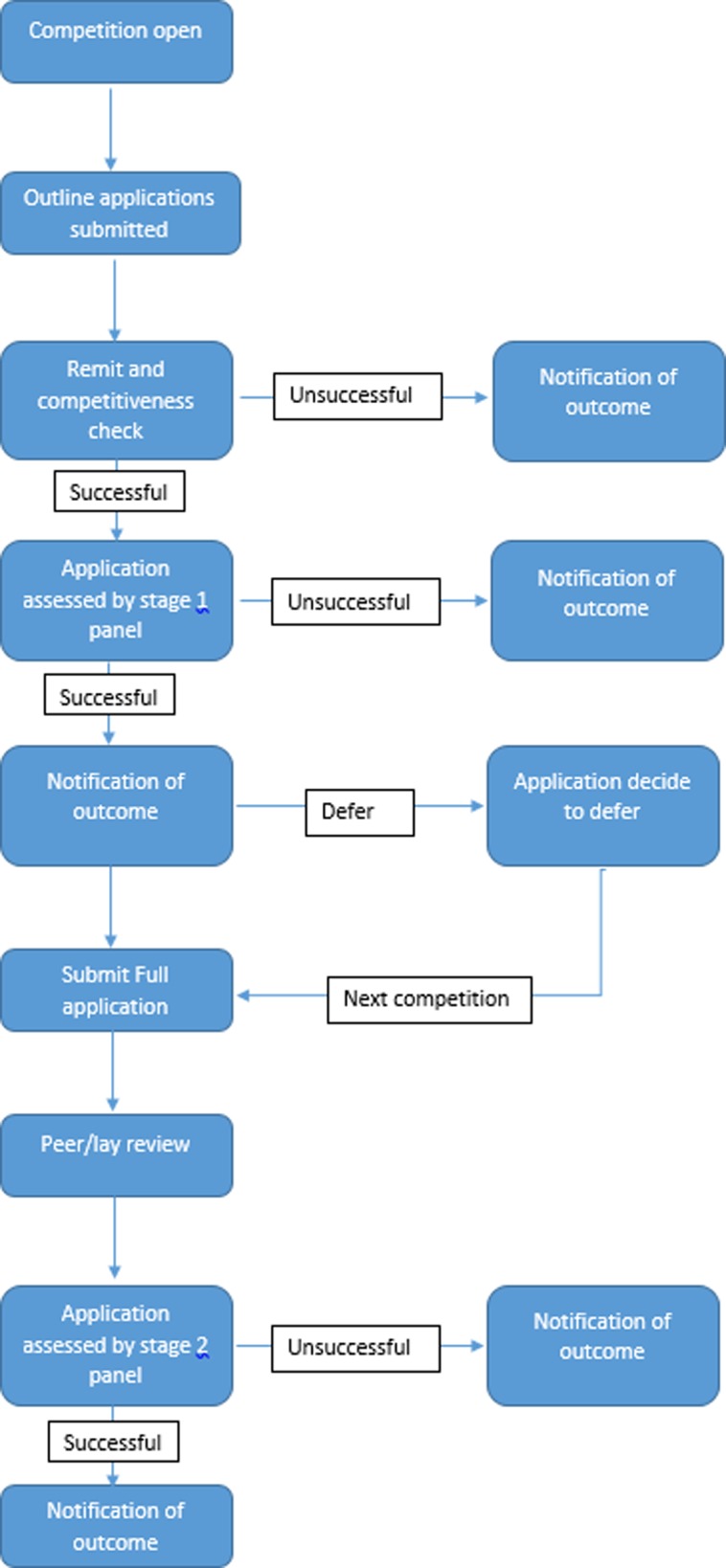
Flowchart of two-stage assessment process.

The two-stage process, as shown in Fig 2, required all applicants to complete an outline application form. All outline application forms submitted under the two-stage process are reviewed for remit and competitiveness by the RfPB staff and panel chairs. This is a relatively swift review to triage out any applications which are clearly out of the RfPB remit and/or incomplete or uncompetitive. All outline applications are then assessed by a RfPB panel to decide whether to invite to submit a full application form to stage 2. The outline assessment by the panel is based on the following broad criteria:

• the relevance and importance of the research question to patients and the National Health Service
• the appropriateness of endpoints (e.g. are the outcome measures patient centred)
• the amount of improvements required for a competitive Stage 2 full application
• the appropriateness of the method
  • Will the proposed methods achieve the stated aims?
  • Does the proposed method imply an unwarranted burden for patients?

The panels then score each application using a 1 to 10 range (with 10 being the highest) and use the mean score to decide which should be invited to submit a full application.

All full applications were then sent for external peer and lay review. The peer reviewers are typically methods and topic experts who did currently not sit on an RfPB panel. The RfPB panel then assesses the full applications, factoring in comments from the external peer and lay reviewers, to decide which to recommended for funding. When making the decision to recommend an application for funding the RfPB panels use the following broad criteria:

• scientific quality of the proposal
• the likely impact on health and social care and the potential for patient benefit in the short to medium-term
• the value for money provided by the proposal

The panels then score each application using a 1 to 10 range (with 10 being the highest) and use the mean score to rank the applications and decide which applications to recommend for funding.

The mandatory sections of the outline form have a collective word limit of (up to) 4,500 words and applications are usually between 15 to 20 pages in total. The mandatory sections of the full form have a collective word limit of (up to) 15,000 words and applications range between 40 to 80 pages in total.

#### Assessment of cost efficiencies of the new process

*Peer review time*. The people asked to conduct peer review are a mixture of academics, clinicians, practitioners, methodologists, and other experts within relevant topic areas. They are typically experienced members of their field and generally fall into the mid to senior career pathway. While there is no average peer reviewer a Clinical Lecturer would be an uncontroversial example. Therefore, we used a Clinical Lecturer salary scale to estimate the cost savings of peer review. Taking the midpoint of a clinical lecturer salary from the University of Oxford pay scale[17] we have assumed the average clinical lecturer salary is £43,247 for a 37.5 hour week. This salary cost has been used to estimate the cost saving by using the hourly cost (£22.18) and the estimated time to complete a peer review (3 hours) for the mean number of peer reviews required by the one and two stage processes.

*Lay review costs*. Lay reviewers are paid for their review. INVOLVE guidelines indicate that lay reviewers are usually paid £100 for their review of an RfPB application form, depending on the size of the application form [18]. This figure was then then used to calculate the cost of lay review for both one and two stage processes based on the mean number of lay reviewers required by each process.

*Panel member time*. The panel meeting is a full day and the programme estimates it takes panel members at least one day to read the applications, meeting paperwork and prepare for the meeting. We assumed each panel has 15 expert members, each member spends one day preparing for and one day attending the panel meeting, and that a clinical lecturer (with a salary of £43,247 working a 37.5 hour week) accurately reflects an average panel member. Therefore, we used the hourly salary cost of £22.18 per panel member to estimate costs associated with requiring two fewer panel meetings per funding round using the two-stage process.

*Programme staff time for peer review*. The programme analysed time taken to complete peer review for an application and it is estimated at 4.45 of staff time hours per application. This includes various processes such as:

• reading through relevant sections of application form to identify which peer reviewers may be needed
• sourcing appropriate peer reviewers
• entering peer reviewer details in the management system
• sending initial invites to peer reviewers
• sending initial invite reminders to peer reviewers
• allocating peer reviewers on the management system
• dispatching invites to peer reviewers on the management system
• sending reminders and chasing peer reviewers
• validating submitted peer reviews
• sending acknowledgement emails to peer reviewers
• sending peer reviewers notification of application outcomes.

The average salary cost of the staff members performing peer review is £27,000 based on a 37.5 hour week. We used the average hourly salary cost (£13.85) to estimate the costs for completing peer review for both one and two stages, using the mean number of reviews required.

*Panel meeting costs*. Part of the change to a two-stage process involved reducing the number of regional panels from 10 to 8 based on the rationale that the two-stage process can manage more applications and therefore less panel assessing capacity was needed. The average cost of running a panel is estimated to be £5,437. These costs include venue hire, travel accommodation and meals for RfPB staff and panel members (when needed), fees paid to lay panel members (typically £300 per lay member per meeting[19]), chair honoraria costs and any other operational expenses related to panel meeting attendance.

Therefore, the cost of holding one panel meeting, under both the one and two stage process is estimated at £5,437.

## Results

We consider data from sixteen funding rounds all together, half from the period when the one-stage process was in operation (with a mean number of 114 applications per funding round) and the other eight from after the funding programme changed to a two-stage process (with a mean number of 136 applications per funding round). We present comparative data from before and after the change describing the effects for the funder, the research applicants, the external peer reviewers, the panel members, and science.

### 1. How much longer did the two-stage process take?

**Fig 3:**
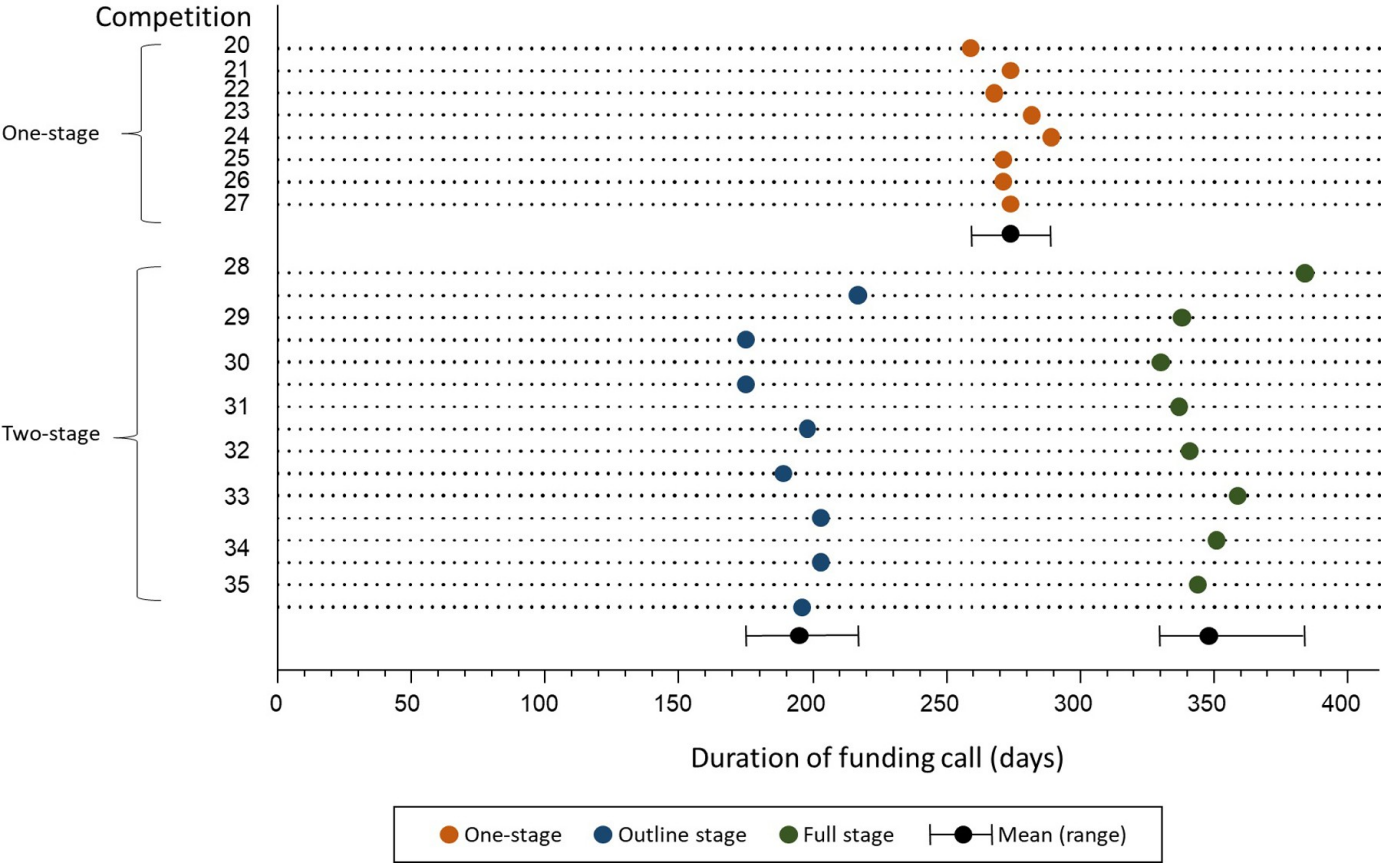
Funding round durations.

As shown in Fig 3, the one-stage application process had a mean (SD) duration of 274 (9) days from funding round close to notification of outcome; the two-stage process had a mean (SD) duration of 348 (17) days between funding round close and outcome at stage 2, which is significantly longer than the one-stage process (mean difference (95% CI): 74 (59.9 to 89.1) days; P<0.001). Under the two-stage process the applications that were declined at the outline stage received feedback at a mean (SD) of 195 (5) days, which is significantly earlier than the one-stage commissioning process (mean difference (95% CI): 79 (66 to 92) days; P < 0.001). This means that a substantial amount of applicants received their final outcome with feedback quicker under the two-stage process when compared to the one-stage process. It is worth noting that the first funding round using the two-stage process is a slight outlier because it was purposefully operated longer than needed whilst the programme adjusted to the new application process.

### 2. Do the external peer review scores suggest there has been any change in the quality of the science?

A total of 1219 applications were submitted to the RfPB between Competition 20 and Competition 35 that underwent external peer/lay review. The number of reviewers for each application ranged from 3 to 10 with mean being 5 reviewers. Almost all applications had one public reviewer. One application received comments from two public reviewers. The mean (SD) of the average score for each application by assessment process and type of reviewers are summarised in Table 1. When developing the two-stage application process it was believed that the quality of the stage 2 applications might improve due to the applicants receiving feedback from the stage 1 panel. By addressing this feedback, the stage 2 application should be more competitive and receive higher external peer and lay review scores, scored using a range of 1 to 10 (with 10 being the highest). The external peer/lay reviewers provide a score which reflects the overall quality of the proposal covering areas such as relevance of the research question, appropriateness of the methodology and trajectory to patient benefit. Results from the mixed effect model analysis suggested that there was an evidence of interaction between process and type of reviewers (peer or lay) (P = 0.014). This means there was evidence of a modest difference in the overall reviewer scores between two-stage and one-stage process (estimated mean difference (95% CI) [two vs one-stage]: 0.36 (0.22 to 0.49); P < 0.001) but no evidence of difference was found between the processes in the lay reviewer scores (estimated mean difference (95% CI): 0.04 (-0.20 to 0.28); P = 0.74).

**Table 1:** Mean and standard deviation of mean peer and lay review scores.

Assessment process	Mean peer reviewer score	Mean lay reviewer score
One-stage, mean (SD) [n^a^]	6.46 (1.20) [819]	6.74 (1.97) [819]
Two-stage, mean (SD) [n^a^]	6.82 (1.17) [400]	6.78 (1.84) [400]

^a^ Number of applications

### 3. Has the number of applications submitted to the programme changed and was there an effect on success rates?

**Fig 4:**
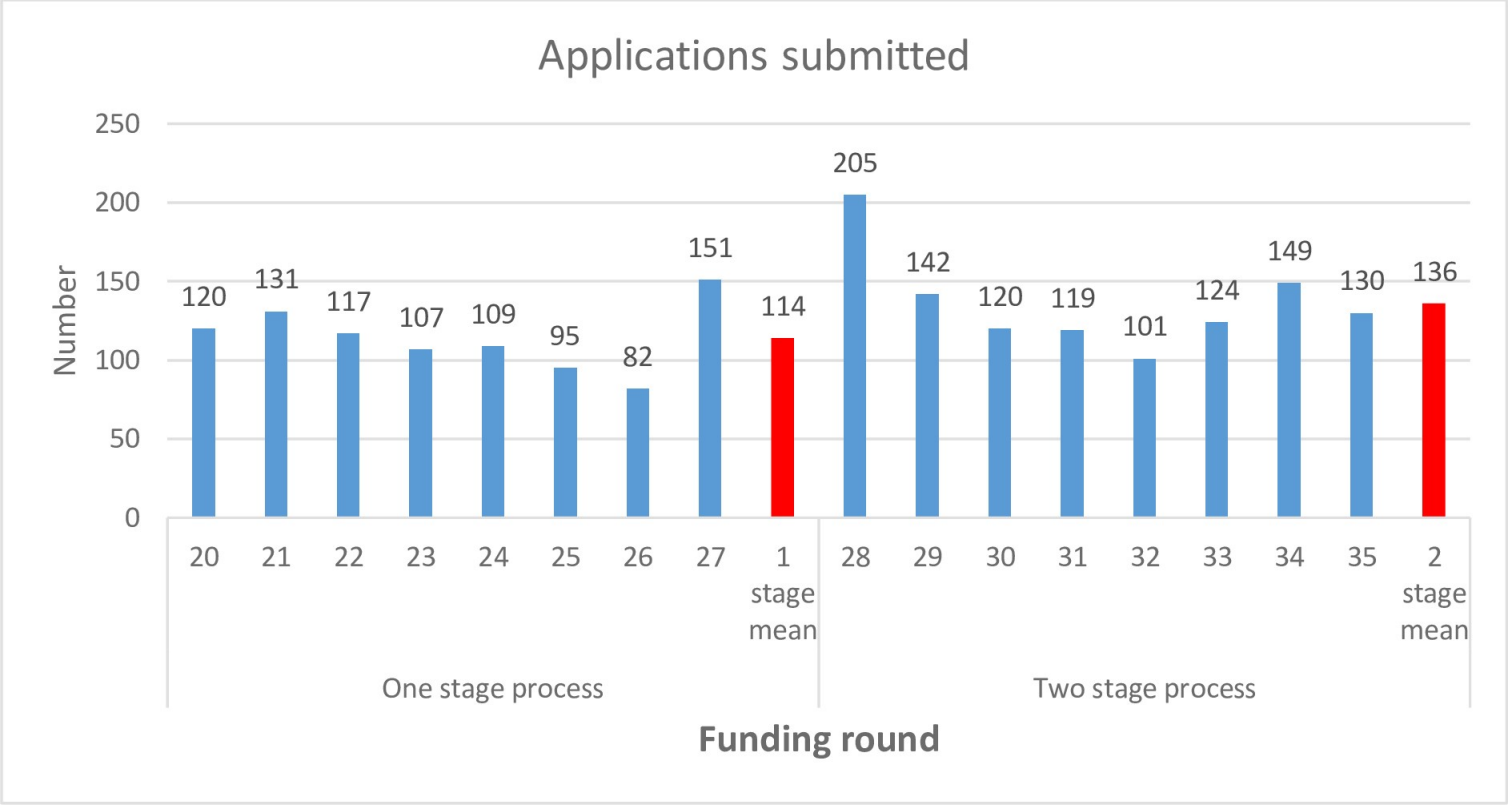
Total applications submitted between funding rounds 20 and 35.

The two-stage application process was associated with an increase in the applications submitted to the programme with a mean of 22 more applications per funding round. As can be seen in Fig 4, there are two funding rounds where there was a spike in applications, the last funding round using the one-stage process (funding round 27) and the first funding round using the two-stage process (funding round 28). This was believed, based on anecdotal feedback, to be due to a rush of applicants submitting to funding round 27 before the changes were introduced. The high number of applications to funding round 28 was not unexpected because new innovations to funding programmes often attract a higher number of applicants.

As can be seen in Table 2, the overall success rate remained consistent between the one-stage and two-stage processes at 18% even though on average 22 more applications were submitted under the one-stage process. This is due to more applications (4 more, on average) being funded under the two-stage process compared to the one-stage process. The success rate of externally peer reviewed application increased from 21% under the one-stage process to 50% under the two-stage process which aligned with RfPB’s ambitions to fund approximately 50% of applications invited to submit a full application at stage 2. However, the mean success rate of applications invited to submit a full application from an outline application was 38% when the RfPB had envisaged it would be approximately 50%.

**Table 2:** Total applications and success rates.

	Funding Round	Total applications submitted	Total applications passing remit/competitiveness checks (%)	Number of applications invited to submit full application (two stage process only) (%)	Applications funded	Success rate of externally peer reviewed applications (%)	Overall success rate (%)
1 stage process	20	120	89 (74)	n/a	17	19	14
	21	131	106 (81)	n/a	23	22	18
	22	117	105 (90)	n/a	25	24	21
	23	107	98 (92)	n/a	23	23	21
	24	109	103 (94)	n/a	23	22	21
	25	95	91 (96)	n/a	16	18	17
	26	82	79 (96)	n/a	18	23	22
	27	151	148 (98)	n/a	23	16	15
	**1 stage mean**	**114**	**102 (89)**	**n/a**	**21**	**21**	**18**
2 stage process	28	205	205 (100)	62 (30)	30	48	15
	29	142	140 (99)	57 (41)	23	40	16
	30	120	118 (98)	47 (40)	20	43	17
	31	119	119 (100)	45 (38)	25	56	21
	32	101	101 (100)	45 (45)	21	47	21
	33	124	123 (99)	48 (39)	25	52	20
	34	149	149 (100)	53 (36)	30	57	20
	35	130	124 (95)	43 (35)	28	65	22
	**2 stage mean**	**136**	**135 (99)**	**50 (38)**	**25**	**50**	**18**

### 4. How many peer and lay reviewers were required?

Under the two-stage application process external peer review only occurred for applications which progressed to a full stage 2 proposal. Therefore, despite receiving a mean of 22 more applications under the two-stage process a mean of 215 fewer external peer reviews were required per funding round, as shown in Table 3. This reduced the burden on the funder’s staff in sourcing reviewers as well as the research community (who are not paid for peer review).

**Table 3:** Number of reviewers required and obtained (external peer and lay reviewers).

	Funding round	Number of applications requiring peer and lay review	Number of peer reviews obtained (mean per application)	Number of lay reviews obtained
1 stage process	20	89	358 (4.0)	89
	21	106	463 (4.4)	106
	22	105	422 (4.0)	105
	23	98	421 (4.3)	98
	24	103	410 (4.0)	103
	25	91	348 (3.8)	91
	26	79	327 (4.1)	79
	27	148	632 (4.3)	148
	**1 stage mean**	**102**	**423 (4.1)**	**102**
2 stage process	28	62	252 (4.1)	62
	29	57	266 (4.7)	57
	30	47	186 4.0)	47
	31	45	169 (3.7)	46
	32	45	197 (4.4)	45
	33	48	201 (4.2)	48
	34	53	219 (4.1)	53
	35	43	171 (4.0)	43
	**2 stage mean**	**50**	**208 (4.2)**	**50**

It is hard to estimate how much time a peer review takes to complete and it will vary due to many factors such as size and scale of the project, number of pages of the application and complexity of the work. Some funders estimate that an external review could take between an hour and half a day to complete[20]. RfPB would, if asked, suggest that a peer review of one application might take approximately 3 hours to complete. Therefore, it is estimated that, per funding round, the time required to conduct peer review for the one-stage process took 1,269 hours and the time required to conduct peer review for the two-stage process took 624 hours; therefore, a potential mean saving of 645 hours of peer reviewer time could be saved per funding round.

Each application that was externally peer reviewed, under both processes, also received a lay review from a patient/lay member of the public. As can be seen in Table 3 the two-stage commissioning process reduced the review burden on lay reviewers by a mean of 52 per funding round. INVOLVE estimates that a lay reviewer who reviews 3 applications takes between eight to ten hours[18]. Therefore, the two-stage process potentially saves between 139 and 173 hours of lay reviewers’ time per funding round.

### 5. Assessment of cost efficiencies of the new process

The specific areas where cost saving have been estimated, per funding round, are as follows:

• Peer reviewer time
• Lay review costs
• Panel member time
• Programme staff time for peer review
• Panel meeting costs

As shown in Table 4, the total cost changed from £148,908 for a one-stage process to £105,342 for a two-stage process demonstrating a cost saving of £43,566 per funding round.

**Table 4:** Summary of cost differences between one-stage and two-stage processes.

Tasks	One-stage process total costs	Two-stage process total cost	Difference
Peer reviewer time	£28,146	£13,840	£14,306
Lay review costs	£10,200	£5,000	£5,200
Panel member time	£49,905	£39,924	£9,981
Programme staff time for peer review	£6,287	£3,082	£3,205
Panel meeting costs	£54,370	£43,496	£10,874
Total	£148,908	£105,342	£43,566

Some of these costs are direct savings to the funder, such as less staff time required to complete peer review. Other costs savings are indirect and estimated based on the time saved to others in the assessment process, such as peer reviewers and panel members.

Table 5 summarises the advantages and disadvantages of the one-stage and two-stage processes in relation to the objectives being assessed.

**Table 5:** Summary of advantages and disadvantages of two-stage process.

Improvement area	Indicator	Beneficiaries	1 stage process	2 stage process	Outcome
Overall timeframe	Time between application submitted and applicants notified of outcome	Applicants	Mean 274 days	Mean 348 days	Two stage process 74 days longer
Quality of science	External peer and lay review scores	Funder, Applicants	Peer review mean: 6.46	Peer review mean: 6.82	Two stage process saw peer review scores increase by 0.36 but no evidence for difference for lay reviews
			Lay review mean: 6.74	Lay review mean: 6.78	
Number of peer and lay reviews	Volume of peer and lay reviewers required	Peer and lay reviewers, Funder	Peer review mean: 423	Peer review mean: 208	Two stage process requires mean of 215 fewer peer reviews and mean of 52 fewer lay reviews
			Lay review mean: 102	Lay review mean: 50	
Number of applications submitted	Volume of applications submitted	Applicants, Funder	Mean 114	Mean 136	Two stage process increases mean applications by 22
Cost efficiencies	Cost of operating process	Funder, Peer reviewers (who are not paid for reviews) and their employers	-	Cost savings due to requiring fewer panel meetings, lay reviews and time saved by peer reviews and funder staff	Two-stage process saves approximately £43,566 per funding round

## Discussion

We have looked at the consequences of the change from a one-stage to two-stage process for funders, applicants and science. For an additional 74 days in the total length of the application process a number of substantial benefits in other areas were obtained via the two-stage process. However, it is worth noting that not all applications took an additional 74 days to receive their outcome since 62% of outline applications do not progress to full stage 2 applications. Researchers who are not invited to submit a full proposal discover the outcome of their submission on average 79 days earlier.

Despite receiving a mean of 22 more applications under the two-stage process the overall success rates remained constant at 18%. This contrasts with the findings of the National Science Foundation study[6] which showed overall application success rates dropped from 14%-20% to 8%-9%. However, the National Science Foundation study contained a significant higher absolute number of applications and the change to a two-stage process led to a greater percentage increase (28%) of applications compared to RfPB’s 16% increase. While RfPB was able to achieve its aim of funding approximately 50% of full applications at stage 2 it invited a smaller percentage (38%) to submit a full application at stage 2 than the approximately 50% envisaged. Fewer applicants received any external reviews but all receive feedback from the 15–20 member panel, following the discussion. Had the panels invited poor quality applications to stage 2 then we might have expected the mean external peer review scores to be lower than under the one-stage process. Instead, they were higher. A potential risk of using the RfPB panel to assess outline applications without external reviewers (who would typically be topic specific expert), is that the panel may lack the necessary expertise. However, the combined knowledge of the panel members, who are mainly methodologists and clinicians with considerable experience of research design, invariably meets the requirements for the decision. If in doubt, the panels would always have the flexibility to invite a full application which would then be externally peer reviewed by topic experts.

More applicants pass the remit and competitiveness checks under the two-stage process. Under the one-stage process the remit and competitiveness checks may have been more cautious due to panel assessment workload and the need for immediate external reviews. Applicants declined at this stage also received minimal feedback, essentially a letter stating the project was out of remit or not competitive and that it would not be assessed any further. A benefit of more applications progressing past these checks under the two-stage process meant that every stage one applicant receives panel feedback. Typically this is three to six specific feedback points which provide a better view on whether an improved version of the application might be successful in a future competition. Therefore, the two-stage process enables more applications to be assessed by a panel and receive feedback.

External peer reviewer scores are a proxy of application quality and tend to reflect the funder’s specific assessment criteria, which for RfPB are the trajectory to patient benefit and the rigour of the methods. If we take the external peer review scores as an indicator of the quality of the science, there is a small but significant increase in the scores. We think that the increase in peer reviewer scores may be due to applicants making improvements to the study following panel feedback at stage one, thereby increasing the scientific quality of the full application. There was no evidence for an increase in the lay review scores. This may suggest that improvements relate more to technical and methodological aspects of the application. Additional work to examine if the two-stage process leads to higher quality of applications could look at specific impact outcomes/metrics, however with RfPB this would need larger numbers and longer follow up. We also hope that the quality of research proposals and/or the applicants ability to translate the findings into patient benefit is improving over time, which could interfere with a before and after design such as ours.

One of the major benefits of the two-stage process was the reduction of the number of peer and lay reviewers required amounting to an average of 215 (peer) and 52 (lay) per funding round, which typically comprises 100–150 applications reviewed and 20–30 funded, nationally. This reduction is estimated to save 645 hours (peer) and between 139 and 173 hours (lay) of the reviewers’ time respectively per funding round. This is time saved which could be better used for other research activity.

There is also a clear increase in the volume of applications submitted under the two-stage process which may reflect a more proportionate amount of researcher effort and because the two-stage process offers researchers the chance to address panel feedback at outline stage. Under the one-stage process the amount of work to submit an application for a £350k award was not much different to applications to other NIHR programmes for multi million pound awards. The adoption of the two-stage process not only leads to efficiencies for RfPB but brings RfPB in line with the rest of the NIHR programmes. The introduction of the new process appears to have halted the downward trend of application being submitted to the programme.

There are a number of areas where the two-stage process demonstrated cost efficiencies, some of which are realised directly from the mechanics of the two-stage process (such as savings to peer/lay review), and other savings such as the two-stage process allowing the programme to reduce 10 regional panels to eight. RfPB reduced 10 regional panels to eight, partly due fewer applications being submitted under the one-stage process and based on the assumption that the time required to assess outline applications under the two-stage process would be quicker. Therefore, despite having two fewer panels RfPB is able to assess more applications. Another benefit of the two-stage process was that it allowed greater flexibility. For example, applicants invited to stage 2 but who could not address the outline stage feedback in the time allocated (about 6 weeks) could defer to the following funding round. Approximately four applications defer each funding round indicating that this flexibility is useful for some applicants. We did not systematically collect feedback on the change to a two-stage process however, anecdotally, applicants’ feedback on the change has been positive.

This research adds to the existing evidence base on peer review processes[3–7,11] that shows there are ways to manage funding processes in a more efficient manner that is not detrimental to the quality of the research funded. Research funders should regularly review their funding processes to ensure that they are fit for purpose and reduce burden, and waste, to those involved in the processes.

### Strengths and limitations

This review only looks at applications from one funding programme but it covers a large number of applications from a geographically diverse set of applicants and a wide range of topic areas and disciplines. The operational issues discussed are likely to be relevant to many contexts beyond health research, especially those who are considering setting up a project grant scheme that anticipates a high volume of applications. However, it should be noted that other research funders may have different priorities and other research programmes may have different remits and selection criteria and therefore some of the findings may not be generalizable.

There may also be some unintended consequences. Stage 1 panels assessing outline applications do not have the benefit of external peer and lay review so members may score cautiously and cluster around the mid-range meaning potentially strong or weak applications may be inappropriately rejected or invited to full application.

## Conclusions

A two-stage application process comprising outline and full application stages, when used for assessing research projects, offers greater benefits in terms of cost and efficiency savings and greater flexibility for applicants compared to a one-stage full application process. Research funders and research programmes which currently operate a one-stage assessment process should consider the benefits of adopting a two-stage process.

## Supporting information

S1 Table (XLSX)
